# Implementing WHO PEN in primary health in Moldova: a qualitative evaluation of barriers, enablers, and lessons for scale-up

**DOI:** 10.1186/s12875-026-03252-2

**Published:** 2026-03-11

**Authors:** Virginia Salaru, Yelena Tarasenko, Angela Ciobanu, Tiina Laatikainen, Diana Chiosa, Angela Anisei, Zinaida Alexa, Maria Muntean, Ghenadie Curocichin

**Affiliations:** 1 Department of Family Medicine, Nicolae Testemițanu State University of Medicine and Pharmacy, Chișinău, Republic of Moldova; 2 Department of Biostatistics, Epidemiology and Environmental Health Sciences, Jiann-Ping Hsu College of Public Health, Georgia Southern University, Statesboro, USA; 3 World Health Organization, Regional Office for Europe, Copenhagen, Denmark; 4 University of Eastern Finland, Kuopio, Finland; 5 Health Services Quality Management Department, Ministry of Health, Chișinău, Republic of Moldova; 6 Timofei Moșneaga Republican Clinical Hospital; President, Association of Endocrinologists of the Republic of Moldova, Chișinău, Republic of Moldova

**Keywords:** WHO PEN, noncommunicable diseases, primary health care, implementation, qualitative research, LMIC, framework analysis

## Abstract

**Background:**

The WHO Package of Essential Noncommunicable (PEN) disease interventions is promoted to strengthen NCD prevention and management in primary health care (PHC), particularly in low-resource settings. Moldova initiated PEN implementation in 2017. Prior quantitative monitoring showed mixed performance across sites.

**Objective:**

To identify practical strategies to improve implementation and inform national scale-up.

**Methods:**

We conducted a qualitative study (June–August 2018) in five family medicine centers purposively selected for prior PEN performance (two high, one medium, two low). Two trained interviewers per site conducted semi-structured, face-to-face interviews with clinicians. Interviews were audio-recorded, transcribed verbatim (Romanian), anonymized, and analyzed using the framework analysis approach by a six-member team. Findings were discussed iteratively with the evaluation team.

**Results:**

Twenty-eight participants were interviewed (15 doctors, including five managers and 13 nurses). Participants valued PEN for structuring consultations and clarifying team roles. Implementation barriers included staff shortages and workload, duplicate documentation, limited ongoing training (especially for risk communication and motivational interviewing), inconsistent use of cardiovascular risk to guide treatment, referral bottlenecks, and patient-level barriers (costs, limited preventive care use among working-age men, migration). Enablers included joint doctor-nurse training, clear role delineation, simple embedded tools (checklists), local reorganization of patient flow, and leadership engagement.

**Conclusions:**

To sustain impact during scale-up, national efforts should streamline documentation, institutionalize refresher training focused on counseling and risk-based care, address access and referral gaps, and deliver community outreach to under-reached groups. These actions can strengthen PHC and advance national NCD goals.

**Supplementary Information:**

The online version contains supplementary material available at 10.1186/s12875-026-03252-2.

## Contributions to the literature

- This study provides one of the first qualitative analyses of WHO PEN implementation in primary health care in an LMIC in the WHO European Region.
- By complementing routine monitoring data with qualitative insights, this study informs strategies for scaling up WHO PEN and similar NCD interventions in low-resource settings.
- The study identifies multilevel barriers and enablers, spanning providers, systems, and patients, that shape integration of cardiovascular risk prevention into primary care.
- The findings demonstrate how leadership, workflow adaptation, and inter-professional collaboration can support implementation under resource constraints.

## Background

Noncommunicable diseases (NCDs) remain a major public health challenge globally, accounting for more than half of the global disease burden and placing increasing pressure on low- and middle-income countries (LMICs) [1, 2]. Within the WHO European Region, countries with the highest rates of cardiovascular diseases (CVD) often have the weakest and least resourced health systems, characterized by underdeveloped primary health care (PHC) services [3, 4].

The Republic of Moldova, an LMIC in the WHO European Region, faces one of the highest burdens of cardiovascular mortality, with CVD responsible for nearly half of all deaths. The prevalence of major NCD risk factors remains high: in 2021, 34.4% of adults had hypertension, 29.9% were current tobacco users, 63.2% were current alcohol drinkers, 9.1% were physically inactive, and 63.9% were overweight [5]. Responding to this growing burden, the Moldovan health system prioritized strengthening PHC as the first point of contact for prevention, early detection, and long-term management of NCDs.

To advance these goals, the Ministry of Health launched implementation of the WHO Package of Essential Noncommunicable (PEN) Disease Interventions for PHC in 2017. The WHO PEN provides a cost-effective, evidence-based set of protocols for the prevention, diagnosis, and management of major NCDs in resource-limited settings. It comprises two main clinical protocols: PEN Protocol 1, focused on preventing heart attacks, strokes, and kidney disease through integrated management of hypertension and diabetes; and PEN Protocol 2, which emphasizes health education, counseling, and behavioral risk modification [5].

Beginning in June 2017, PEN protocols 1 and 2 were piloted in 20 PHC facilities across Moldova [6]. A three-day introductory training for all facility staff was conducted by national trainers, followed by ad hoc supportive supervision visits by members of the national PEN implementation team. A quantitative evaluation conducted by WHO 12 months after implementation (June 2017–June 2018) demonstrated the feasibility of using simple algorithms and routine data to improve detection and control of CVD risk factors in PHC settings, while revealing variation across sites (e.g., improved detection but weaker risk scoring) [7, 8]. Our qualitative study examined provider-, system-, patient-, and crosscuttingfactors explaining these differences, identified barriers and enablers reported by PHC teams, and drew practical implications for the national scale-up of WHO PEN in Moldova.

## Methods

### Study design and setting

This qualitative evaluation complemented a broader mixed-methods assessment of PEN implementation and was designed as a pragmatic qualitative evaluation embedded within the national PEN pilot. The evaluation was guided by an applied, interpretive approach aimed at understanding how and why PEN protocols were implemented in routine PHC practice, rather than testing a specific theory. The study focused on PHC facilities participating in the national PEN pilot and was conducted between June and August 2018, approximately 12 months after the introduction of PEN protocols. Detailed descriptions of the PEN pilot design, participating facilities, and quantitative performance indicators have been published previously, including the evaluation protocol by Collins et al. [6], and are referenced here to provide contextual background for the present analysis.

### Participant selection

Five PHC facilities were purposively selected from the national PEN pilot to capture variation in prior quantitative performance. Facility performance was assessed using routine monitoring data comparing baseline values with results from the first year of PEN implementation, as reported in previously published evaluations [7, 8]. Performance indicators included documentation of cardiovascular risk factors and diagnoses, selected process indicators (e.g., blood pressure measurement, smoking status recording, SCORE documentation, and prescribing practices), and outcome indicators related to blood pressure control. Based on these indicators, facilities were ranked, and two higher-performing, one medium-performing, and two lower-performing facilities were selected to reflect diverse implementation experiences. Participants were selected based on their professional roles and minimum 12-month involvement in PEN implementation, ensuring adequate experience with the intervention. All individuals invited to participate agreed to take part.

### Data collection

At each facility, two trained interviewers (from a total of five interviewers involved in data collection) conducted semi-structured, face-to-face interviews with a purposive sample of clinicians (i.e., family doctors, including facility managers, and nurses). The semi-structured interview guide was developed by members of the national PEN implementation team using a Delphi exercise to identify questions most relevant to the objectives of the qualitative evaluation. Following initial development, the guide was pilot-tested with two family doctors and two nurses from PHC facilities that did not participate in the study, and feedback was used to refine question wording and clarity. The final guide included core questions applicable to all participants, as well as role-specific questions for managers, family doctors, and nurses. The interview guide was developed and administered in Romanian, the working language of the study.

Interviewers used a flexible guide that allowed them to explore core topics while adapting to emerging themes (Suppl. 1 in English and Suppl. 2 in Romanian). Data collection continued until data saturation was reached, when no new themes emerged. Interviews were audio recorded with participant consent and transcribed verbatim. Transcripts were not returned to participants for comment or correction due to time constraints. Interviewers also made brief field notes during and after interviews. Interviews lasted between 30 and 60 min. Interviews with facility managers were generally longer (closer to 60 min), reflecting broader discussion of organizational and system-level processes. Interviewers were public health professionals involved in national PEN implementation, but were not involved in supervision, monitoring, or evaluation of the participating facilities. Only the interviewer and participant were present during interviews. No repeat interviews were conducted. Interviewers received training in qualitative interviewing prior to data collection.

### Researcher reflexivity

Given that interviewers were members of the national PEN implementation team, the research team explicitly considered the potential influence of insider positionality, power dynamics, and social desirability on data collection and interpretation. Interviewers’ familiarity with the implementation context facilitated informed probing and understanding of local practices; however, it may also have influenced participant responses. To mitigate these risks, interviewers emphasized confidentiality, voluntary participation, and the independence of the research from performance assessment. During analysis, reflexive discussion within the multidisciplinary research team was used to critically examine assumptions, challenge interpretations, and ensure that findings reflected participants’ perspectives rather than implementation expectations.

### Data analysis

Transcribed interviews were analyzed using the framework analysis approach [9]. Six members of the multidisciplinary research team were involved in the analysis and collaboratively developed the initial coding framework. Following transcription, the multidisciplinary analysis team familiarized themselves with the data and developed an initial coding framework informed by the study objectives, interview questions, and anticipated domains corresponding to provider-, system-, and patient-level factors. During data familiarization, additional codes emerged inductively from participants’ accounts. Through iterative rounds of coding and regular team discussion, the coding framework was reviewed, refined, and organized into a coherent analytic structure that incorporated both a priori and data-driven concepts.

Each analyst independently coded and charted transcripts using the agreed framework, with themes compared across facilities and professional groups (managers, doctors, nurses). All interviews were anonymized, and participants were identified only by role (e.g., *Manager 1*, *Doctor 2*, *Nurse 3*). Data were organized and coded using Microsoft Excel, which enabled matrix charting consistent with the framework analysis approach. Codes were iteratively grouped into categories, which were subsequently synthesized into overarching themes consistent with the framework analysis approach.

Discrepancies in coding or interpretation were addressed through regular team discussions. When differences arose, analysts compared coded excerpts, revisited the data, and discussed interpretations until agreement was reached. Where needed, senior members of the research team facilitated discussion to support consensus and ensure that interpretations were grounded in the data and aligned with the study objectives.

### Ethical Considerations

Ethical approval was obtained from the Nicolae Testemitanu State University of Medicine and Pharmacy Ethics Committee (Approval No. 83, 31 May 2017). The Ministry of Health authorized the evaluation before data collection. The study was conducted in accordance with the ethical principles of the Declaration of Helsinki. In each facility, interviewers met with the head of the institution to explain study objectives and obtain permission. All participants received written and verbal information about the study and provided written informed consent prior to participation. Confidentiality and voluntary participation were emphasized throughout.

## Results

### Overview

Twenty-eight participants (15 doctors and 13 nurses) from five PEN pilot clinics were interviewed. Five of the doctors were clinic managers, two had partial managerial responsibilities for coordinating several general practitioners, and two nurses held senior roles as chief nurses, which included managerial and coordination responsibilities. Consistent with their functional roles, these participants were asked both nurse- and manager-specific interview questions. Most respondents (24/28) had attended the initial WHO-supported PEN training in 2017, while four had received informal or on-the-job instruction.

Although facilities differed in quantitative performance, the qualitative analysis revealed broadly similar patterns of barriers and enablers across sites; therefore, results are presented thematically rather than stratified by performance category.

**Fig. 1:**
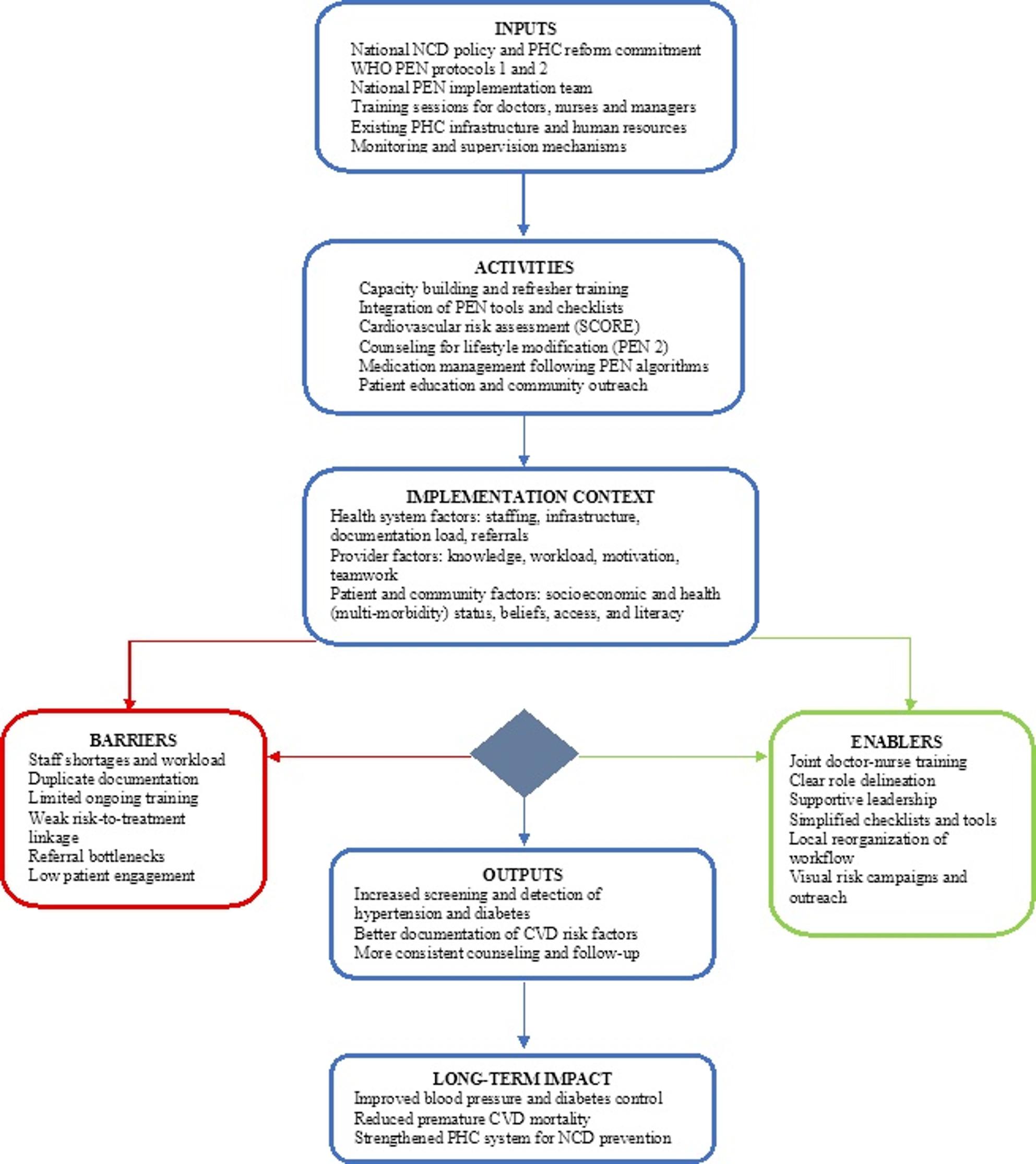
Logic model illustrating the implementation of WHO PEN protocols in primary health care in the Republic of Moldova

The framework analysis generated eight overarching themes reflecting key barriers and enablers to the implementation of WHO PEN protocols within PHC settings in the Republic of Moldova. These themes operated across three interrelated levels – provider, system, and patient – capturing both organizational and behavioral dynamics shaping practice change. Fig. 1 presents a logic model illustrating how these factors interact within the PEN implementation process. Within each level, specific factors constrained or facilitated implementation, from workforce capacity and infrastructure to patient engagement and community health promotion. Illustrative quotations from family doctors, nurses, and managers highlight diverse perspectives across facilities (Table 1).

**Table 1:** Summary of barriers and enablers to WHO PEN implementation in the Republic of Moldova

Level	Analytic Theme	Barriers	Enablers	Illustrative Quote(s)
Provider	Workforce capacity and training	Staff turnover; lack of systematic induction; uneven refresher opportunities; reliance on self-training.	Joint doctor–nurse training; shared tools/language; peer support among nurses.	*“Once we have all been trained*,* we all know how to apply the PEN protocols.”* (Manager 2) *“We support each other—if someone forgets*,* the others remind.”* (Nurse 3)
Provider		Ambiguous task division; variable/experiential use of risk tools; inconsistent interpretation.	Managerial leadership; joint training; clearer delegation and teamwork.	*“The doctor and I (nurse)*,* we both assess it.”* (Nurse 4) *“We started paying more attention to all the details.”* (Nurse 2)
System	Health system and infrastructure	Heavy workloads; staff shortages; fragmented patient flow; equipment/space constraints; limited specialists.	Local workflow redesign (separate rooms, screening areas); managerial initiative.	*“We started by separating family doctor’s work from that of the nurse.”* (Manager 5) *“If I had enough time and space for each patient*,* I wouldn’t find it difficult to help them.”* (Nurse 7)
System	Documentation and workflow processes	Lack of time; duplicate/triplicate records; incomplete forms for risk factors; under-documentation.	Staff motivation; openness to new tools/checklists; intention to improve processes.	*“We often lack time.”* (Nurse 1) *“We try to record everything correctly—it’s important for us*,* too.”* (Nurse 6)
Cross-cutting (Provider and System)	Delivery of clinical and preventive care	Mechanical/indicator-driven SCORE use; lab delays; tariff changes; counseling time constraints.	Peer feedback and collective review; PEN 2 structure; workflow adaptation.	*“At the beginning*,* the cardiovascular score was slightly lower… we tried to assess the cardiovascular risk more accurately.”* (Manager 3) *“We do the math*,* then we give it to the doctor*,* and he is the one to tell the patient their risk.”* (Nurse 10)
Patient	Socio-demographic characteristics	Migration; poverty; episodic attendance; limited access to specialists and healthy foods.	Older adults’ more frequent attendance enabling monitoring.	*“The patient comes and undergoes screening but refuses to go to the doctor for consultation.”* (Nurse 2) *“Older patients are more disciplined—they come regularly for control.”* (Doctor 4)
Patient	Psycho-cognitive particularities	Low perceived risk; denial/stigma around alcohol/tobacco; poor adherence, especially in younger/asymptomatic patients.	Empathetic communication; personalized feedback.	*“Patients say they don’t need it because they are healthy.”* (Manager 5) *“We try as calmly as possible to tell them about smoking and drinking.”* (Nurse 7)
Patient	Accessibility to services and risk assessmen**t**	Multiple visits required; transport costs not covered; preference for unscheduled same-day care; waiting times.	Same-day testing; streamlined patient pathways.	*“They must pay two or three visits to the health facility for primary evaluation.”* (Interview summary) *“We try to do everything in one visit so the patient doesn’t have to return.”* (Nurse 8)

### Provider-level factors

#### Workforce capacity and training

Most respondents (24 of 28) had been trained by national PEN trainers during WHO-led modules in June–July 2017. Participants found the modules useful and valued training in mixed groups of nurses and family doctors, which fostered shared language and tools for patient evaluation.

*“Once we have all been trained*,* we all know how to apply the PEN protocols.”* (Manager 2).

Staff turnover and the absence of systematic induction training for new employees created knowledge gaps; late-arriving staff learned through self-training or by observing colleagues. All interviewees emphasized the need for continuous training in PEN use and in integrated approaches to behavior change among patients with noncommunicable diseases. Frequency and content of training varied widely across facilities. Nurses often relied on peers for support, while doctors preferred self-training.

*Barrier*: Staff turnover and uneven refresher opportunities limited consistency.

*Enabler*: Mixed-team training and peer learning strengthened shared understanding and collaboration. 

#### Attitudes and ownership toward PEN implementation

All participants confirmed familiarity with the PEN protocols and recognized their mandatory role in clinical practice.

*“I think protocols must be followed.”* (Manager 4).

Use of PEN varied from daily to “particular situations” (e.g., high-risk patients). Clinicians favored protocols that were clear and practical: PEN 1 was viewed as more doctor-focused and PEN 2 as more nurse-oriented.

*“We started paying more attention to all the details.”* (Nurse 2).

Providers described learning to move beyond numeric tables toward fuller clinical appraisal.

*“We initially didn’t pay attention to the additional factors that could influence the risk; we only calculated the risk using the tables; therefore*,* the risk was underestimated; after the evaluations*,* we were informed and are now more careful in assessing the cardiovascular risk.”* (Manager 3).

Misinterpretation of tools and informal workarounds were reported. Some nurses used the BMI chart thinking it was a risk table, while some doctors estimated risk “from experience” rather than using the SCORE charts.

*“I can orient myself without having to calculate.”* (Doctor 1).

Unclear task division sometimes caused duplication or omission.

*“The doctor and I (nurse)*,* we both assess it.”* (Nurse 4).

Facilities led by managers trained in PEN showed stronger team support and clearer task delegation.

*Barrier*: Ambiguous task division and inconsistent interpretation of tools.

*Enabler*: Leadership engagement and prior joint training encouraged accountability and teamwork.

### System-level factors

#### Health system and infrastructure

Heavy workloads and staff shortages were universal, with some clinicians covering multiple roles and rural populations, requiring extra travel.

*“The doctor serves 6000 population.”* (Manager 1).

*“Our doctors have a catchment population over 3000. They are very overloaded due to lack of time.”* (Manager 5).

PEN increased workload, especially for nurses.

*“Most of the activities … set out by PEN … are carried out by the nurse.”* (Manager 1).

New tasks such as bilateral blood-pressure measurement, diabetic-foot exams, and lifestyle assessments, alongside other reforms, added time pressure. Staff linked reluctance to change to poor recognition of prevention importance, inefficient organization (e.g., triage not used), ambiguous task division, and fragmented patient flow.

*“The patient goes straight to their family doctor*,* without going through the triage room.”* (Nurse 5).

Documentation burden was heavy; duplicate entry across systems consumed time.

*“We keep dual records.”* (Nurse 5).

New tools (e.g., AUDIT and Fagerström testing) increased workload and reduced counseling time. Opinions on the prevention checklist were mixed: some saw it as burdensome; managers valued it for audit optimization.

### Space and equipment constraints

Crowded or under-equipped offices and distant screening rooms limited fidelity to PEN protocols. Ophthalmoscopy was rarely performed due to lack of equipment and training.

*“I cannot afford to have a separate screening room; we do not have space.”* (Manager 2).

*“If I had enough time and space for each patient*,* I wouldn’t find it difficult to help them.”* (Nurse 7).

Some facilities reorganized rooms to improve workflow.

*“We started by separating family doctor’s work from that of the nurse.”* (Manager 5).

Limited collaboration with laboratories and specialists delayed access.

*“We don’t have a lab technician; we’ve been requesting one for many years*,* but no one comes.”* (Manager 2).

*“The lack of specialists is a very important factor.”* (Manager 5).

Patient referrals to medical facilities in other localities generated added costs for patients and facilities, also impacting timely provision of examinations or consultations.

*Barrier*: Staffing and infrastructure constraints and referral delays.

*Enabler*: Local workflow redesign and managerial initiative mitigated bottlenecks.

#### Documentation and workflow processes

Time constraints made it difficult to address all patient issues; counseling time was often sacrificed.

*“We often lack time.”* (Nurse 1).

*“They are very overloaded due to lack of time … when no one opens the door during their consultations.”* (Manager 5).

This focus on acute problems reduced preventive counseling capacity. Managers proposed greater nurse involvement, but many nurses lacked skills for motivational interviewing.

Medical forms lacked fields for risk documentation, impeding monitoring.

*“I don’t know where to write it down; it’s not in the form.”* (Nurse 9).

Some staff avoided sensitive terms when recording alcohol or smoking history.

*“We use abbreviations*,* sometimes on purpose*,* in order not to offend the patient.”* (Manager 2).

Certain procedures were performed but not documented because of time pressure.

*“I think they measure it on both arms*,* but simply do not write it down in the form.”* (Nurse 3).

Documentation of risk-reduction interventions was inconsistent, and trend supervision was absent. For example, an obese patient was recommended a healthy lifestyle, but the continuity of counseling and the proper follow-up of patient was missing. Multimorbidity further increased paperwork and coordination demands, as multiple risk factors and conditions must be tracked across forms and visits, contributing to under-documentation of counseling and trend supervision.

*Barrier*: Incomplete documentation and time limits disrupted continuity. Fragmented forms and parallel registries are poorly suited to multimorbidity.

*Enabler*: Staff motivation and openness to new tools supported improvement.

### Cross-cutting: provider and system

#### Delivery of clinical and preventive care

### Cardiovascular risk assessment

Participants discussed varied approaches to cardiovascular risk assessment under PEN, reflecting both strong role division and occasional ambiguity in responsibility. Nurses often conducted initial calculations, while doctors verified and communicated results to patients.

*“I think the doctor should do it because he is more knowledgeable.”* (Nurse 8).

*“Nurses are our right hand … They take care of everything … and only when there are complex patients … we do it together.”* (Manager 2).

*“We do the math*,* then we give it to the doctor*,* and he is the one to tell the patient their risk.”* (Nurse 10).

Risk estimation was sometimes mechanical, performed mainly to meet requirement for the national performance indicators. Quality control was weak, but feedback later improved accuracy.

*“At the beginning*,* the cardiovascular score was slightly lower … Following your evaluations*,* we were informed … We started discussing collectively and tried to assess the cardiovascular risk more accurately.”* (Manager 3).

Cholesterol testing delays impeded same-visit risk calculation and required multiple visits, and some patients declined repeat visits.

*“Many times*,* they don’t have time to go to the triage room or to the lab.”* (Nurse 7).

Risk discussion and trend tracking were inconsistently documented. Only some doctors mentioned evaluating trends when using risk-reduction interventions. Flaws were found in risk interpretation and communication to patients by both family doctors and nurses; hence, patients were often not told the SCORE result nor the need to reduce it. However, family doctors confirmed that recommendations were adjusted based on the cardiovascular risk.

*Barrier*: Mechanical use of charts and fragmented quality oversight, delayed cholesterol testing requiring multiple visits, and gaps in documenting and communicating risk and trends.

*Enabler*: Peer discussion and feedback improved accuracy and confidence; when completed, risk-informed recommendations were applied to the case. Team-based task sharing (nurse-led checks with physician confirmation) can streamline visits for multimorbid patients.

### Clinical examinations and investigations according to PEN

Providers described both logistical and procedural challenges in carrying out routine examinations and laboratory investigations. While basic tests were accessible, external laboratory contracts and resource shortages introduced delays and inequities.

*“The investigation is free*,* but the travel costs.”* (Manager 5).

*“I have listed it for you: we are short of doctors*,* or they are on a leave; when we send them to the Republican Polyclinic*,* they tell us there that our endocrinologist should see them first*,* but he is not there because he is also entitled to take leave or attend continuous medical education. … The lack of specialists is a very important factor.”* (Manager 5).

Outsourced labs, reagent shortages, and tariff changes reduced access. Routine procedures like diabetic-foot exams and bilateral BP measurement were inconsistently applied. Sensitive tests (AUDIT, Fagerström) were often skipped because of discomfort or unreliable patient responses.

*“We do not ask them how much alcohol they drink per day.”* (Manager 3).

*Barrier*: Lab delays, cost, and provider discomfort with sensitive discussions.

*Enabler*: Workflow adaptation and delegation improved feasibility in some clinics.

### Medication-based treatment

Discussions about pharmacological treatment revealed tensions between clinical guidelines and practical constraints. While protocols guided prescribing behavior, financial barriers and patient adherence challenges persisted.

*“The patient accepts the treatment because*,* if he feels pain*,* he takes it … but in statins the effect is not felt … if they were compensated their acceptability would grow.”* (Doctor 5).

*“Our patient is afraid of medications*,* claiming they are tired of them*,* that there are too many and will affect their liver or pancreas*,* and then they come with hypertensive crisis*,* or a trivial exacerbation*,* which could be catastrophic for a diabetic patient.”* (Doctor 3).

Statin use was low due to lack of reimbursement and weak adherence. Treatment often followed protocols but not individualized risk.

*Barrier*: Cost and limited risk-based prescribing.

*Enabler*: Standardized national protocols maintained treatment coherence.

### Lifestyle counseling and health promotion

Counseling patients on behavioral risks such as smoking and alcohol use remained one of the most sensitive and least standardized components of PEN implementation. Providers cited lack of time, training, and educational materials as major obstacles to effective communication.

*“Firstly*,* I do not feel very comfortable to tell them to stop drinking.”* (Nurse 4).

*“Doctors currently deal with the disorders and not with their prevention.”* (Manager 4).

*“I … have an issue with counseling … we try as calmly as possible and around the bush to tell them about smoking and drinking.”* (Nurse 7).

Time, privacy, and skill limitations hindered motivational interviewing. Educational materials were scarce, and public health campaigns were limited.

*“We deal with the consequences … Education should start from nursery*,* kindergarten*,* school … we will not succeed without joint efforts of the community and public administration.”* (Doctor 2).

*Barrier*: Counseling discomfort, lack of materials, and absent public campaigns.

*Enabler*: PEN 2 structured tools and team motivation provided a foundation for expansion.

### Patient-level factors

#### Patient socio-demographic characteristics

Participants emphasized that patients’ social and demographic contexts shaped care access. Older adults were the main users of preventive care, while younger working-age men rarely sought services. Migration and economic hardship disrupted continuity of care.

*“The patient comes and undergoes screening but refuses to go to the doctor for consultation.”* (Nurse 2).

The presence of comorbidities complicated management, requiring coordination with absent specialists or social workers and often resulting in late presentation and poor disease control.

*Barrier*: Poverty, migration, and episodic visits undermined continuity. Multimorbidity increased complexity of care and the need for coordinated follow-up.

*Enabler*: Older adults’ frequent attendance allowed steady monitoring.

#### Patient psycho-cognitive particularities

Participants frequently described patients’ attitudes, beliefs, and levels of health literacy as key influences on prevention and adherence. Many patients underestimated their risk, denied unhealthy behaviors, or felt stigmatized when confronted about tobacco or alcohol use.

*“Patients say they don’t need it because they are healthy.”* (Manager 5).

*“Patients come with reproaches: why did you write in my card that I abuse?”* (Nurse 6).

*“We have found patients of a very young age with high blood pressure*,* but they regard it as a joke.”* (Manager 2).

*Barrier*: Low health literacy and stigma-related perceptions.

*Enabler*: Empathetic communication and tailored feedback improved openness.

#### Accessibility to health services, including risk assessment

Accessibility challenges limited full PEN implementation. Participants noted that patients often had to make several visits for screening and laboratory results, which discouraged follow-up and continuity of care.

*“They must pay two or three visits to the health facility for primary evaluation.”* (Interview summary).

Multiple visits and travel costs discouraged follow-up. Patients preferred unscheduled same-day services with minimal waiting.

*Barrier*: Repeated visits and transport costs restricted access.

*Enabler*: Same-day testing and streamlined patient flow were promising solutions.

### Summary across levels

Barriers to PEN implementation in Moldova spanned workforce shortages, infrastructure limits, documentation gaps, cost barriers, and patient disengagement. Enablers included inter-professional training, managerial leadership, structured tools from both PEN 1 (risk stratification and treatment algorithms) and PEN 2 (counseling and behavior-change tools), local workflow innovations, and strong provider motivation to strengthen prevention despite systemic constraints.

## Discussion

This qualitative study explored the experiences of PHC providers and managers during the implementation of WHO PEN protocols in the Republic of Moldova. The findings provide an in-depth understanding of the contextual, organizational, and patient-level factors that influenced the uptake and sustained use of PEN interventions. While previous quantitative analyses demonstrated the feasibility of PEN implementation and improvements in detection and control of selected cardiovascular risk factors [6–8], our study highlights several structural, procedural, and behavioral barriers that continue to constrain full integration of NCD prevention and management in PHC practice.

Overall, the implementation of PEN protocols was perceived by family doctors, nurses, and managers as both useful and feasible in daily practice. The protocols offered clear guidance, supported task-sharing between doctors and nurses, and helped structure consultations. These findings mirror evidence from other LMICs, where PEN protocols have strengthened PHC service delivery by providing standardized algorithms and promoting inter-professional collaboration [3, 4, 10]. However, despite general acceptance, participants described substantial challenges related to human resources, training, and infrastructure. High workloads, staff shortages, and paperwork demands limited the time available for comprehensive risk assessment and counseling. The increased responsibilities assigned to nurses, though beneficial for task redistribution, were not always matched with sufficient training or supervision.

Communication of cardiovascular risk to patients emerged as a critical gap. Many providers felt uncertain about interpreting or explaining risk scores, leading to mechanical use of charts rather than personalized counseling. This aligns with findings from PEN implementation studies in Bhutan and Myanmar, where limited training and weak patient engagement were major barriers to effective risk communication [10, 11]. Similar to patterns described in the recent systematic review of WHO PEN implementation across 15 countries [12], Moldova’s experience confirms that workforce training, motivation, and consistent feedback are among the most decisive factors for successful integration of PEN protocols into PHC routines. The Moldovan experience further suggests that investment in motivational interviewing, patient-centered communication, and continuous professional development could help translate risk assessment into sustained behavioral change and improved adherence.

At the system level, infrastructural and procedural constraints, such as delays in cholesterol testing, incomplete integration of laboratory and referral systems, and duplication in documentation, undermined continuity of care. As also reported in Kyrgyzstan and other LMICs implementing WHO PEN [10–13], challenges related to laboratory capacity, equipment, and coordination between PHC and higher-level facilities were recurrent system-level barriers. In Moldova, however, local managerial leadership, peer feedback, and adaptive workflow redesign were important enablers that helped mitigate some of these obstacles. These findings reflect international evidence that decentralized leadership, supportive supervision, and context-specific adaptation are essential for embedding new clinical protocols [10].

Patient-related challenges further influenced implementation outcomes. Socioeconomic hardship, migration, and low health literacy limited consistent attendance and adherence to preventive care. Younger adults often perceived themselves as healthy and declined screening or treatment, while older patients tended to attend more regularly, enabling better monitoring. Similar barriers were reported in other LMICs implementing WHO PEN, particularly regarding the role of patient trust, stigma, and affordability of care [10–14]. Despite these challenges, Moldova demonstrated several positive enabling conditions, including public coverage of essential laboratory tests and medicines, that helped offset some cost-related barriers identified in other settings.

An important interpretive insight emerging from the findings concerns the nature of the barriers described by participants. Rather than reflecting outright resistance to WHO PEN protocols, many challenges appeared to represent pragmatic adaptation to existing system constraints. Providers generally expressed positive attitudes toward PEN and recognized its clinical value, yet described difficulties related to workload, documentation demands, resource limitations, and patient engagement. These patterns suggest that implementation tensions were driven less by opposition to the intervention itself and more by the practical realities of integrating new protocols into already strained clinical workflows. This distinction is consistent with implementation research indicating that apparent “barriers” often reflect structural misalignment, competing demands, and capacity limitations rather than deliberate resistance. Understanding these dynamics is critical for policy and program design, as solutions may require organizational and systemic adjustments rather than strategies focused solely on provider motivation or compliance.

The logic model presented in Fig. 1 synthesizes the implementation pathways identified through the empirical findings and illustrates how barriers and enablers interact across provider, system, and patient levels. Key barriers, including workforce shortages, high workload, documentation burden, and referral bottlenecks, constrained the consistent application of WHO PEN protocols in routine practice. At the same time, several enabling factors identified across facilities, such as joint doctor-nurse training, supporting managerial leadership, and local reorganization of patient flow, mitigated these constraints. For example, in response to limited human resources and increasing workload, some facility managers redistributed tasks between doctors and nurses and introduced structured patient pathways to reduce duplication of activities. Expanding the role of nurses in health education and risk assessment allowed more efficient use of consultation time and improved feasibility of preventive care delivery. By grounding the logic model in observed practice, the findings demonstrate its value as an evidence-informed tool to guide adaptation and scale-up of WHO PEN implementation in resource-constrained PHC settings.

The core PEN implementation methodology was standardized across participating facilities, reflecting national program design and training structures. However, variations in organizational context, resource availability, staffing patterns, and patient population characteristics influenced how PEN protocols were operationalized in practice. These contextual differences likely contributed to variation in quantitative performance indicators, while not fundamentally altering the types of barriers and enablers identified. Across facilities, participants described similar structural and systemic challenges, suggesting that broader health system constraints played a more prominent role than facility-specific implementation strategies. This pattern aligns with implementation research emphasizing that standardized interventions are often mediated by local context rather than implemented uniformly.

Although participating facilities were purposively selected to capture variation in prior quantitative performance, the qualitative analysis was not designed to explain performance-related differences. Instead, the study aimed to identify cross-cutting barriers and enablers shaping PEN implementation across primary health-care settings. The findings indicate that many implementation challenges were experienced similarly across facilities, suggesting that structural and systemic factors, such as workforce constraints, financing limitations, and patient engagement difficulties, play a more prominent role than facility-specific performance characteristics. Variations observed in the data were more strongly associated with professional roles than institutional ranking, with doctors, nurses, and managers emphasizing different aspects of implementation consistent with their responsibilities. This role-based differentiation underscores that PEN integration represents not only a clinical intervention but also an organizational and behavioral adjustment within primary care practice.

As with many implementation studies, interviewer positionality may have influenced how experiences were articulated and interpreted. Because interviewers were members of the national PEN implementation team, participants may have framed their responses in ways they perceived as socially desirable or aligned with program expectations, potentially contributing to a more favorable characterization of PEN implementation. At the same time, interviewers’ familiarity with the implementation context facilitated informed probing and nuanced understanding of local practices. These potential influences were mitigated through reflexive analytic practices, multidisciplinary team interpretation, and triangulation of professional perspectives. Nevertheless, these dynamics should be considered when interpreting the findings.

### Strengths and limitations

This study has several strengths. First, it offers an in-depth, multi-perspective qualitative evaluation of WHO PEN implementation, incorporating the views of family doctors, nurses, and facility managers. PHC facilities were purposively selected from different regions of the Republic of Moldova and represented high-, medium, and low-performing sites based on prior quantitative assessments, allowing exploration of implementation experiences across diverse organizational and geographic contexts. Second, the use of a structured framework analysis approach enabled systematic comparison across provider-, system-, and patient-level factors, supporting transparency and analytic rigor. Third, embedding this qualitative evaluation within a broader mixed-methods assessment strengthened interpretation and policy relevance. Additionally, following completion of data analysis, findings were presented and discussed in a joint validation meeting with representatives from all ten PEN pilot institutions, providing an opportunity to confirm the credibility and practical relevance of the results.

Several limitations should also be acknowledged. As a qualitative study, the non-probability sampling strategy and sample size were not intended to support generalizability; rather, the findings aim to provide transferable insights applicable to similar PHC settings. The evaluation focused on provider and system perspectives and did not include patient experiences, which represent an important dimension of health-care quality and should be addressed in future research. Finally, interviews were conducted by members of the national PEN implementation team, which may have influenced participant responses or interpretation through social desirability or power dynamics. To mitigate this risk, interviewers were not involved in supervision or evaluation of the participating facilities, confidentiality was emphasized, and data were analyzed by a multidisciplinary team with iterative discussion and reflexive consideration of potential bias.

Our study builds upon the existing evidence base regarding the implementation of the WHO PEN package for NCDs in LMICs [3, 4]. While previous quantitative research in Moldova confirmed the feasibility of the program [6–8], the present qualitative analysis reveals the operational and behavioral dimensions necessary for sustainable integration of PEN into routine primary care. Together with global evidence [10–14], our findings reaffirm that successful implementation of PEN requires coordinated interventions at multiple levels (provider, system, and patient) and that ongoing training, adequate financing, and local leadership are central to long-term success. Following completion of data collection and analysis, the findings will be shared and discussed with primary care teams and Ministry of Health stakeholders as part of the national scale-up of WHO PEN, allowing implementers to interpret and apply the findings in practice. This feedback loop supports the feasibility and acceptability of the recommendations at the frontline.

## Conclusions

The implementation of WHO PEN protocols in the Republic of Moldova has led to meaningful improvements in primary care practices, particularly in risk assessment and early detection of cardiovascular disease. However, the long-term success of this initiative depends on addressing persistent barriers at provider, system, and patient levels. Simplifying documentation processes, strengthening inter-professional and patient communication, and ensuring adequate staffing and training are critical for sustainability. Expanding access to affordable diagnostics and medications, alongside targeted health education campaigns, can further enhance the reach and effectiveness of PEN interventions and support national NCD management goals.

## Supplementary Information

Supplementary Material 1.

Supplementary Material 2.

Supplementary Material 3.

## Data Availability

The qualitative interview transcripts generated and analysed during the current study are not publicly available because they contain information that could compromise participant confidentiality and institutional privacy. De-identified data may be available from the corresponding author on reasonable request and with permission from the Ministry of Health of the Republic of Moldova. The interview guide and coding framework can be shared on request.

## Abbreviations

WHO: World Health Organization
PEN: Package of Essential Noncommunicable Disease Interventions
NCD: Noncommunicable Diseases
PHC: Primary Health Care
CVD: Cardiovascular Diseases
BMI: Body Mass Index
AUDIT: Alcohol Use Disorders Identification Test
SCORE: Systematic Coronary Risk Evaluation
LMIC: Low–and Middle–Income Countries
