# Supplementary material for: Implementing WHO PEN in primary health in Moldova: a qualitative evaluation of barriers, enablers, and lessons for scale-up
Source: BMC Prim Care. 2026 Mar 11;27:142. doi: 10.1186/s12875-026-03252-2 (PMC13088807; doi:10.1186/s12875-026-03252-2)
Supplement: Supplementary file 2 — Supplementary Material 2. [file 12875_2026_3252_MOESM2_ESM.docx]

**Questionnaire for data collection for qualitative study**

**Basic Questions (for all participants)**

- 1. Please tell me what your role is in this institution?
  2. I have reviewed several patient medical records and prescribed medications, and I would like to discuss some results. The results are aggregated, so we will not discuss specific cases or patients. What do you think about these results? Suggestions based on results (examples)
  3. Do you think we could improve care for CVD and diabetes for our patients? a. Why? b. How?
  4. What could be the challenges (in improving care for CVD and DM)?
  5. How do you think another institution/primary healthcare unit or another person performing similar functions to yours could achieve these changes (to improve care for CVD and DM)?
  6. We believe that PEN Protocols could be useful in your institution. I want to ask you what you think: a) about global cardiovascular risk assessment? b) about the results of using PEN Protocols? c) about the ease of use/complexity of PEN Protocols? d) the usefulness of PEN Protocols in the management of patients with multiple risk factors and/or concomitant pathologies (diabetes and CVD)? e) the usefulness of PEN Protocol No. 2 in counseling patients with risk factors for non-communicable diseases?
  7. If you were to start the pilot implementation of PEN Protocols today, what would you do differently, based on your accumulated experience?
  8. What difficulties do you encounter in the integrated approach to patients with multiple risk factors and/or concomitant pathologies?
  9. Is there anyone else you think I should talk to who might be helpful? I will not tell them that you referred to them, so they will not know if you participated in the evaluation or not.
  10. Would you like to add anything to your answers?

**Additional questions for managers**

1.Discuss whether, or how, the division of tasks (task sharing/delegation) was modified, or how it should have been modified. Was it possible? If yes, how? If no, why? a. How have the roles of your team members changed in the process of applying PEN Protocols? b. How have the role and responsibilities of family doctors changed in the process of applying PEN Protocols? c. How have the role and responsibilities of medical assistants changed in the process of applying PEN Protocols?

1. Do you have a system for scheduling patient visits in your institution (by phone, online)? a. If yes - to what extent is it applied? b. If no - what is used instead?
2. Since the piloting of PEN Protocols, have there been any changes in the patient data recording system for CVD and DM? a. If yes, specifically what and what was their usefulness? b. What other modifications/adjustments are needed?
3. Does your institution have a quality control system/procedures? a. If yes, what stimulated/motivated their implementation/application? b. If yes, what was their usefulness? c. What other modifications/adjustments are needed?
4. For the implementation of PEN Protocols, were adjustments needed in the medical institution's equipment (laboratory, equipment, etc.) or other actions?
5. Would you like to add anything to your answers?

**Additional questions for family doctors**

- 1. How would you explain the term "total cardiovascular risk" to someone?
  2. We are interested in your experience regarding the assessment of CVD risk. Have you used CVD risk assessment in practice? a. If yes, when was the last time you used this assessment? b. If yes, could you describe how you did it? c. If they cannot provide a concrete answer, present an example to verify the correctness of the risk level calculation (Annex 1)?
  3. What is the role of knowing the CVD risk level in the decision-making process regarding patient management?
  4. In your opinion, are there advantages to using CVD risk? a. If yes, which ones?
  5. What are the disadvantages of using CVD risk?
  6. What determines or would determine you to assess CVD risk?

1. What prevents or could prevent you from assessing CVD risk? a. What could help you overcome these impediments?
2. Are you aware of the recommendations regarding the use of the assessed CVD risk level in clinical practice? a. If yes, what do you think about them?
3. How would you communicate a risk to a patient? (Ask the participant to provide a real example or use the scenario provided if the participant cannot provide an example) (Annex 1)?
4. Which group of patients will you assess for CVD risk level? Why?
5. How has your practice changed during the implementation period of PEN Protocols?
6. Have you delegated new functions to the medical assistant since the initiation of PEN Protocols implementation? a. If yes, what are these functions?
7. During the implementation of PEN Protocols, did you need additional support/assistance? a. If yes, what kind?
8. Would you like to add anything to your answers?

**Additional questions for family medical assistants**

- 1. How has the implementation of PEN Protocols changed the care you provide regarding primary prevention and early detection of non-communicable diseases (including treatment and monitoring)?
  2. Describe if and how the division of tasks between doctors and medical assistants has changed in the process of implementing PEN Protocols?
  3. How do you perform each of the following tasks: a. Total CV risk assessment? b. Counseling on lifestyle changes? c. Motivational interviewing? d. Measurement of risk factors (e.g., blood pressure, AUDIT, BMI)?
  4. What support/assistance did you receive from your institution (e.g., from managers, family doctors) for implementing PEN Protocols? a. How did they support you? b. Did they complicate or make implementation more difficult?
  5. Has your confidence in approaching patients with CVD and DM changed? a. How has it changed? b. What are the benefits of these changes? c. What are the disadvantages of these changes?

**Annex 1**

**Clinical case for determining the correctness of cardiovascular risk calculation** Patient Domnica, 67 years old, upon admission reports occipital headache, her father suffered from arterial hypertension and died at the age of 54 from a stroke. She has been smoking since the age of 26. Married. 2 pregnancies, 2 births, one of which with preeclampsia (mentions she was hospitalized with hypertension and edema during pregnancy).

Height 170 cm

Body weight 88 kg

BP 158/98 mmHg

Total cholesterol – 7.8 mmol/l

BMI = 30.45

Waist circumference = 96 cm

Fasting glucose – 6.9 mmol/l
