# Supplementary material for: Implementing WHO PEN in primary health in Moldova: a qualitative evaluation of barriers, enablers, and lessons for scale-up
Source: BMC Prim Care. 2026 Mar 11;27:142. doi: 10.1186/s12875-026-03252-2 (PMC13088807; doi:10.1186/s12875-026-03252-2)
Supplement: Supplementary file 3 — Supplementary Material 3. [file 12875_2026_3252_MOESM3_ESM.docx]

**Chestionar de colectare a datelor pentru studiul calitativ**

**Întrebări de bază (pentru toți participanții)**

1. Spuneți-mi vă rog care este rolul dvs. în această instituție?
2. Am examinat câteva cartele medicale ale pacienților și medicamentele prescrise și aș dori să discutăm despre unele rezultate. Rezultatele sunt agregate, astfel nu vom discuta cazuri concrete sau pacienți specifici. Ce credeți despre aceste rezultate?

Sugestii în baza rezultatelor (exemple)

1. Credeți că am putea îmbunătăți asistența în caz de BCV și diabet zaharat pentru pacienții noștri?

a. De ce? b. Cum?

1. Care ar putea fi provocările (în îmbunătățirea asistenței în caz de BCV și DZ)?
2. Cum credeți ce ar ajuta o altă instituție/unitate de asistență medicală primară sau o altă persoană care îndeplinește funcții similare cu ale dvs. să realizeze aceste schimbări (de îmbunătățire a asistenței în caz de BCV și DZ)?
3. Noi credem că Protocoalele PEN ar putea fi utile în instituția dvs. Vreau să vă întreb ce credeți dvs:
   1. despre evaluarea riscului cardiovascular global?
   2. despre rezultatele utilizării Protocoalelor PEN?
   3. despre ușurința utilizării/complexitatea Protocoalelor PEN?
   4. utilitatea Protocoalelor PEN în conduita pacienților cu multipli factori de risc și/sau patologii concomitente (diabet zaharat și BCV)?
   5. utilitatea Protocolului PEN nr.2 în consilierea pacienților cu factori de risc pentru bolile netransmisibile?
4. Dacă ați începe implementarea pilot a Protocoalelor PEN de astăzi, ce ați face diferit, reieșind din experiența acumulată?
5. Ce dificultăți întâlniți în abordarea integrată a pacienților cu mai mulți factori de risc și/sau patologii concomitente?
6. Ar mai fi cineva cu care credeți că ar trebui să discut și care ar putea să fie de ajutor? Eu nu le voi spune că dvs. v-ați referit la dânșii, astfel ei nu vor cunoaște dacă dvs. ați participat sau nu în evaluare.
7. Ați mai dori să completați cu ceva răspunsurile dvs?

**Întrebări adiționale pentru manageri**

1. Discutați dacă, sau cum s-a efectuat, sau cum trebuia să fie modificată divizarea sarcinilor (partajarea/delegarea sarcinilor). A fost posibil? Dacă da, cum? Dacă nu, de ce?
   1. Cum s-au schimbat rolurile membrilor echipei dvs. în procesul de aplicare a Protocoalelor PEN?
   2. Cum s-a modificat rolul și obligațiunile medicilor de familie în procesul de aplicare a Protocoalelor PEN?
   3. Cum s-a modificat rolul și obligațiunile asistentelor medicale în procesul de aplicare a Protocoalelor PEN?
2. Dispuneți de un sistem de programare a vizitelor pacienților în instituția dvs. (prin telefon, online)?
   1. Dacă da - în ce măsură este aplicat acesta?
   2. Dacă nu – ce este utilizat în loc?
3. Din momentul pilotării Protocoalelor PEN au survenit schimbări în sistemul de înregistrare a datelor pacienților cu BCV și DZ?
   1. Dacă da, care anume și care a fost utilitatea acestora?
   2. Ce modificări/ajustări mai sunt necesare?
4. În instituția dvs aveți un sistem/proceduri de control al calității?
   1. Dacă da, ce a stimulat/impulsionat implementarea/aplicarea acestora?
   2. Dacă da, care a fost utilitatea acestora?
   3. Ce modificări/ajustări mai sunt necesare?
5. Pentru implementarea Protocoalelor PEN au fost necesare ajustări în dotarea instituției medicale (laborator, echipamente, etc) sau alte acțiuni?
6. Ați mai dori să completați cu ceva răspunsurile dvs.?

**Întrebări adiționale pentru medici de familie**

1. Cum ați explica cuiva termenul ”riscul cardiovascular total”?
2. Suntem cointeresați în experiența dvs. privind evaluarea gradului de risc al BCV. Ați utilizat în practică evaluarea gradului de risc al BCV?
   1. Dacă da, când a fost ultima dată când ați utilizat evaluarea acestuia?
   2. Dacă da, ați putea să-mi descrieți cum ați făcut?
   3. Dacă nu poate oferi un răspuns concret, prezentați un exemplu pentru a verifica corectitudinea calculării nivelului de risc (anexa 1) ?
3. Ce rol are cunoașterea gradului de risc al BCV în procesul de luare a deciziei privind conduita pacientului?
4. În opinia dvs., există avantaje în utilizarea riscului BCV?
   1. Dacă da, care?
5. Care sunt dezavantajele utilizării riscului BCV?
6. Ce vă determină sau v-ar determina să evaluați riscul BCV?
7. Ce vă împiedică sau ar putea să vă împiedice să evaluați riscul BCV?
   1. Ce ar putea să vă ajute să depășiți aceste impedimente?

8. Cunoașteți despre recomandările privind utilizarea în practica clinică a gradului de risc al BCV evaluat?

a. Dacă da,ce credeți despre acestea?

1. Cum ați comunica un risc pacientului? (Rugați participantul să vă ofere un exemplu real sau utilizați scenariul oferit dacă participantul nu poate să ofere un exemplu) (anexa 1)?
2. Cărui grup de pacienți veți evalua gradul de risc al BCV? De ce?
3. Cum s-a modificat practica dvs. în perioada de implementare a Protocoalelor PEN?
4. Ați delegat funcții noi asistentei medicale de la inițierea implementării Protocoalelor PEN?
   1. Dacă da, care sunt aceste funcții?
5. Pe parcursul implementării Protocoalelor PEN, ați avut nevoie de suport/asistență suplimentar(ă)?
   1. Dacă da, care anume?
6. Ați mai dori să completați cu ceva răspunsurile dvs?

**Întrebări adiționale pentru asistenții medicali de familie**

1. Cum a schimbat implementarea Protocoalelor PEN asistența pe care o oferiți în ceea ce privește prevenirea primară și depistarea precoce a bolilor netransmisibile (inclusiv tratamentul și monitorizarea)?
2. Descrieți dacă și cum divizarea sarcinilor medicilor și asistentelor medicale s-a schimbat în procesul de implementare a Protocoalelor PEN?
3. Cum realizați fiecare din următoarele sarcini:
   1. Evaluarea totală a riscului CV?
   2. Consilierea privind schimbarea stilului de viață?
   3. Intervievarea motivațională?
   4. Măsurarea factorilor de risc (de ex. tensiunea arterială, AUDIT, IMC)?
4. De care suport/susținere ați beneficiat din partea instituției dvs. (de ex. de la manageri, medici de familie) pentru a implementa Protocoalele PEN?
   1. Cum v-au susținut?
   2. Nu au complicat sau au făcut mai dificilă implementarea?
5. S-a schimbat încrederea dvs. în abordarea pacienților cu BCV și DZ?

- a. Cum s-a schimbat?

b. Care sunt beneficiile acestor schimbări?

c. Care sunt dezavantajele acestor schimbări?

Anexa 1

Caz clinic pentru determinarea corectitudinii calării riscului cardiovascular

Pacienta Domnica, de 67 de ani, la primire spune că are cefalee occipitală, că tatăl ei a suferit de hipertensiune arterială și a decedat la vârsta de 54 de ani de ictus.

Fumează de la vârsta de 26 ani. Căsătorită. 2 sarcini, 2 nașteri, dintre care una – cu preeclampsie (menționează că a fost spitalizată cu HTA și edeme în timpul sarcinii).

Înălțimea 170 cm

Masa corporală 88 kg

TA 158/98 mm Hg

Colesterol total – 7.8 mmol/l

IMC = 30,45

Circumferința taliei = 96 cm

Glicemia *a jeun* – 6.9 mmol/l
